# Our Journal

**Published:** 1871-09

**Authors:** 


### OUR JOURNAL.

Taking into consideration surrounding circumstances, we have no reason to be discouraged in the prosecution of this enterprise. We commenced, and have thus far continued the third series under very unfavorable auspices, pecuniarily. Financially, this country was never, probably, in a more depressed condition. The demands, it is true, are not large, but very general. Capitalists do not find them, separately, of sufficient magnitude to attract their attention, and yet all the surplus means of the South thrown broadcast into the country would barely relieve the trouble.

In this state of money matters it is not expected that any undertaking of the kind will run smoothly at once. Now, however, since the time is at hand when the staple agricultural products will be thrown on the market, with the prospect of more remunerative prices and less outstanding debts to absorb the income than were met with by farmers twelve months ago, a better prospect opens. Whenever money is easy with the farmer it certainly flows into other branches of business, and all are alike relieved from pecuniary embarrassment.

We mention these things to show why we have toiled faithfully and patiently through the summer months, without sufficient return from the "Journal" to meet our liabilities. We labor in hope of a "better time coming," and are determined to make the "Journal" worthy the patronage of physicians throughout the country. In order to do this, we fill much of our space with practical facts, obtained in proceedings of medical societies, clinical lectures, etc., and are making arrangements to secure a corps of contributors not excelled in this or any other country.

A living faith in the success of our undertaking prompts us to make of the "Journal" everything required by the profession; and we shall confidently wait, with patience, for the subscriptions to sustain the work, which will be coming in during fall and winter.

We hope those who receive the "Journal" and approve our efforts to furnish the profession with medical news, will aid us in extending the circulation by sending on the names of *cash subscribers.* This we ask, not as a means of money-making on our part, but that a sufficient amount may be raised from this source to pay for the publication, with which we will be content.

